# An Oncocytic Variant of Papillary Thyroid Carcinoma Mimicking As Metastatic Adenocarcinoma: A Diagnostic Challenge

**DOI:** 10.7759/cureus.48425

**Published:** 2023-11-07

**Authors:** Aishwarya Ray, Sadhana D Mahore

**Affiliations:** 1 Pathology, NKP Salve Institute of Medical Sciences and Research Centre and Lata Mangeshkar Hospital, Nagpur, IND

**Keywords:** immunohistochemistry, histopathology, papillary neoplasm, papillary thyroid carcinoma, oncocytic variant

## Abstract

Papillary thyroid carcinoma is the most common type of thyroid carcinoma, diagnosed on the basis of a predefined set of distinctive nuclear features. There are about 15 known variants of papillary thyroid carcinoma, and the oncocytic variant is not one of the commonly encountered prototypic conventional papillary thyroid carcinoma. We hereby report an unusual case of a 48-year-old woman presenting with thyroid swelling, which proved to be a diagnostic crisis.

## Introduction

An uncommon variety of papillary thyroid neoplasm is its oncocytic variant, the pathological and behavioural features of which have not been adequately characterized [1]. They account for about 1 to 11% of all papillary thyroid neoplasms [2]. They are believed to have originated from oncocytic follicular cells, proven by thyroglobulin immunoreactivity. They depict the traditional papillary arrangement of tumour cells along with classical nuclear features of papillary thyroid carcinoma. However, features such as tumour necrosis or increased mitotic activity are not seen [3]. The pattern of multifocal growth is not a common feature [4]. These tumours have to be distinguished from other oxyphilic thyroid neoplasms because of their different biological characteristics and prognostic implications [4].

## Case presentation

**Figure 1:**
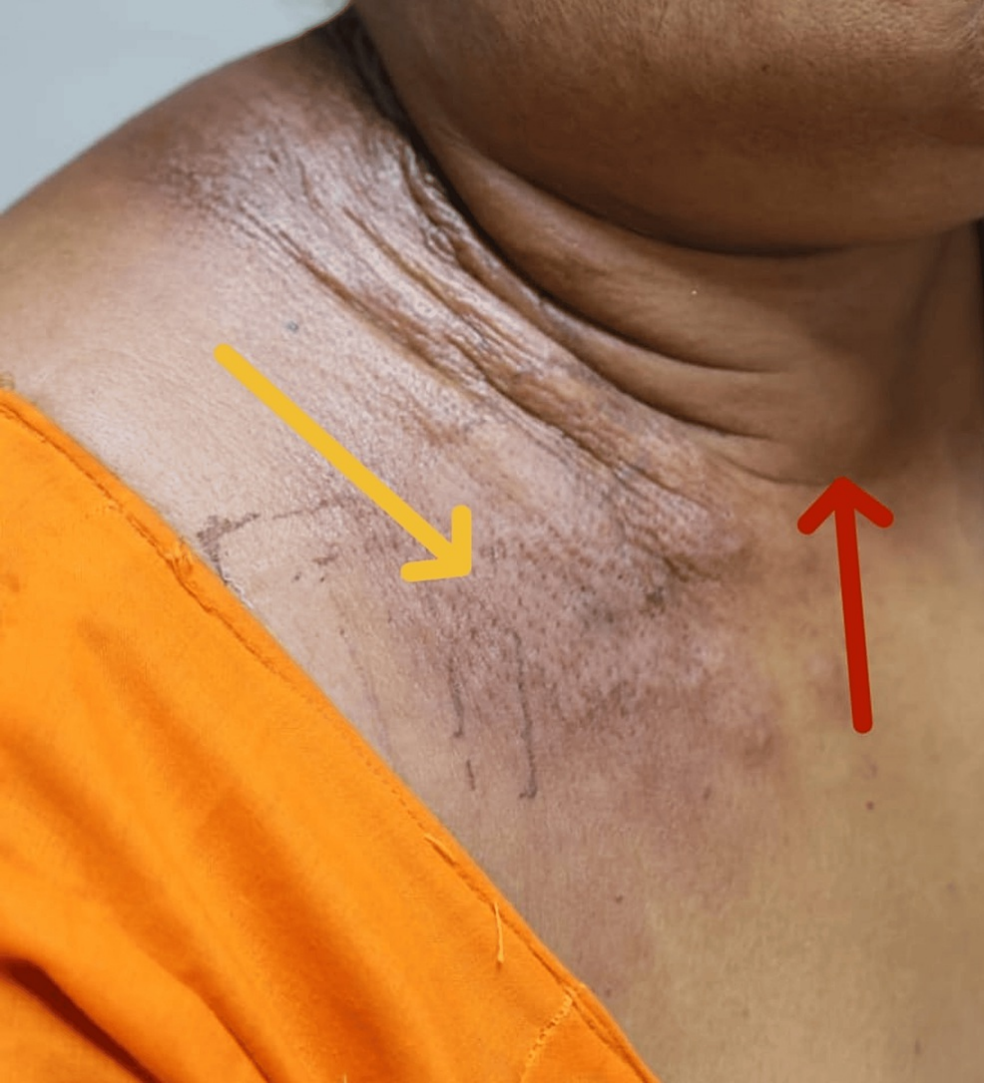
Clinical photograph of thyroid swelling on the anterior aspect of the neck on the right side (red arrow) with cutaneous manifestations along the neck region (yellow arrow).

A 48-year-old woman came in with a three-month history of neck swelling on the right side. She experienced pain and limited neck movement, as well as the development of cutaneous manifestations along the neck region (Figure 1).

Multislice spiral CT showed the thyroid to be diffusely bulky, with multiple hypodense lesions scattered in bilateral parenchyma, suggestive of neoplastic aetiology, along with subcutaneous changes of cellulitis of the overlying skin.

Thorax showed a well-defined heterogeneously enhancing soft tissue lesion of size 2.2 x 2.1 x 3.0 cm along the mediastinal pleura in the apical segment of the right upper lobe with spiculated margins. Multiple subcentimetric and enlarged heterogeneously enhancing lymph nodes were seen in the para tracheal, subcarinal, right hilar and right paratracheal region. The imaging features were likely suggestive of pleural and mediastinal lymph node metastasis.

Fine needle aspiration (FNA) from right cervical swelling revealed scanty to moderate cellularity comprising loose clusters, small sheets and papillaroid fragments of pleomorphic cells having a moderate amount of cytoplasm, high nucleus-to-cytoplasmic (N/C) ratio, granular powdery chromatin and occasional nuclei with grooves. The background showed red blood cells admixed with few lymphocytes. The features were suggestive of metastatic deposits of an epithelial malignancy.

**Figure 2:**
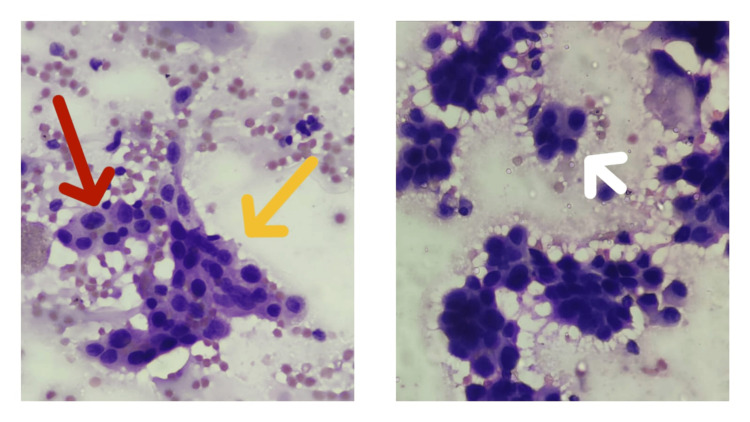
Fine needle aspiration cytology image from thyroid swelling showing papillae, sheets (yellow arrow) and microfollicles (white arrow) of cells having pleomorphic nuclei with/without nucleoli and a moderate amount of cytoplasm (red arrow).

Ultrasonography-guided FNA from the thyroid revealed cellular smears comprising papillae, sheets, a few microfollicles and dispersed cells showing pleomorphic nuclei with nucleoli and a moderate amount of cytoplasm, with occasional grooves. Plasmacytoid cells and mitosis were seen. Fibrous fragments with areas of calcification were also seen. The background showed blood with some lymphoid cells. On the basis of cytological features, the diagnosis of a thyroid neoplasm, most probably papillary thyroid carcinoma, was given (Figure 2).

**Figure 3:**
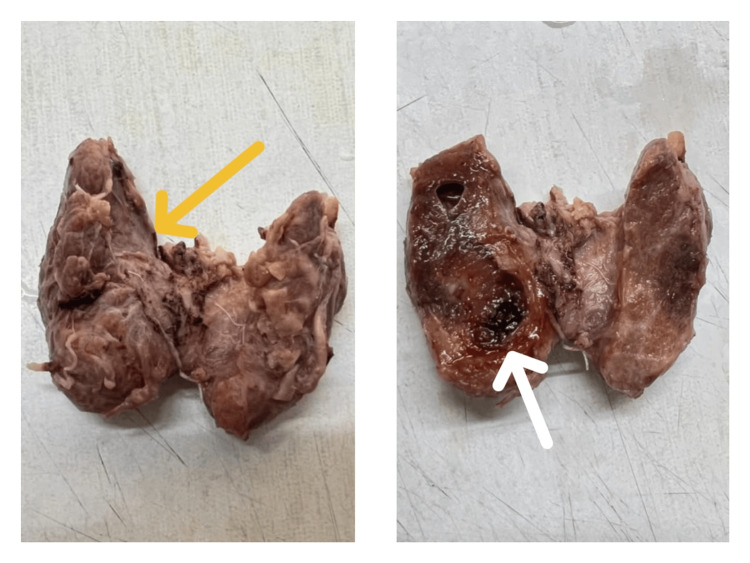
The gross image of the intact specimen of the thyroid (yellow arrow). The cut surface showed nodular, firm, pale to brownish areas with diffuse hemorrhagic areas (white arrow).

The patient underwent total thyroidectomy and an intact specimen of thyroid measuring 5.6 x 4.3 x 1.5 cm was received for histopathological evaluation. On cutting open, both the lobes showed nodular, firm pale, brownish areas with diffuse hemorrhagic areas (Figure 3).

**Figure 4:**
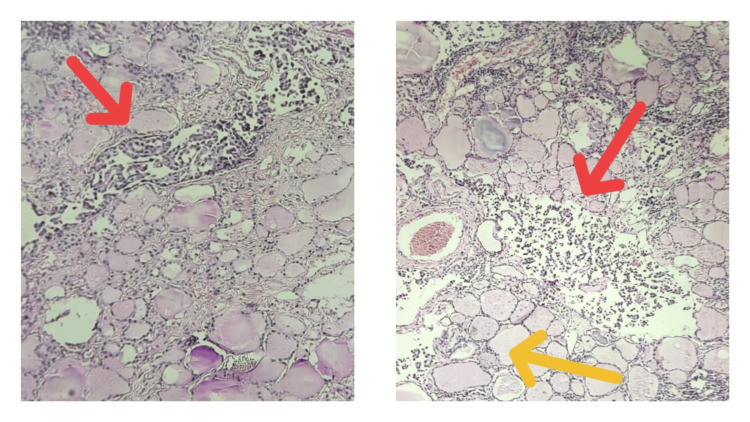
Scanner view (4x) of H&E stained sections showing tumour cells infiltrating between the thyroid follicles (red arrow), with surrounding normal thyroid parenchyma (yellow arrow). H&E: Hematoxylin and eosin

**Figure 5:**
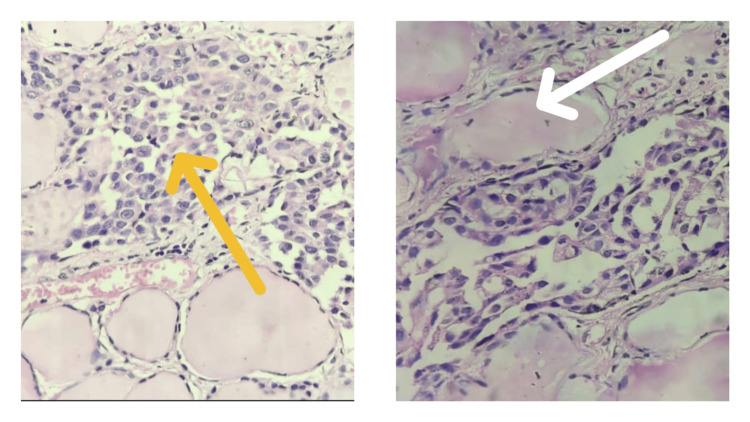
Low power (10x) view of H&E sections showing tumour cells arranged in glandular/acinar pattern. They appear to be mostly dissociated with a few arranged in solid sheets or trabeculae, exhibiting moderate anisonucleosis and pleomorphism (yellow arrow). Adjacent normal thyroid follicles filled with colloids are seen (white arrow). H&E: Hematoxylin and eosin

**Figure 6:**
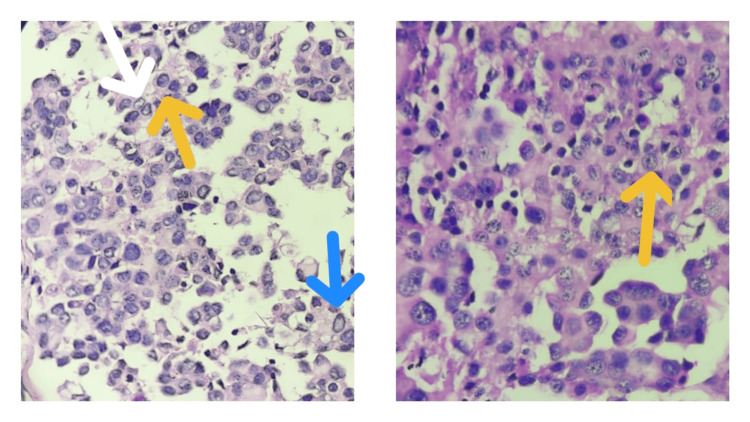
High power (40x) view of H&E stained sections showing round to polygonal tumour cells, having large hyperchromatic nuclei, occasional nucleoli and a scanty amount of cytoplasm. Occasional tumour cells exhibit nuclear grooving (yellow arrows), chromatin clearing (white arrow) along with pseudoinclusions (blue arrow). H&E: Hematoxylin and eosin

On histopathology, sections taken from different areas of the thyroid revealed thyroid follicles having abundant colloid. Tumour cells were seen infiltrating in between the normal thyroid follicles, throughout the whole of thyroid tissue. Tumour cells were round to polygonal, with large hyperchromatic nuclei, with or without nucleoli and a scanty amount of cytoplasm, arranged in glandular and acinar patterns. Cells appeared to be mostly dissociated and some arranged in cords, solid sheets or trabeculae, showing moderate anisonucleosis and pleomorphism. Occasional tumour cells exhibited nuclear grooving and chromatin clearing with pseudoinclusions. A focal collection of lymphocytes and occasional areas of calcification were also seen. Lymphovascular invasion was not seen (Figures 4, 5, 6).

Based on the above histopathological features, it was difficult to arrive at an unequivocal diagnosis. Thereupon, a primary malignancy of the thyroid, most probably a variant of papillary carcinoma thyroid, was considered as one of the differentials. Metastatic deposits of adenocarcinoma in the thyroid could not be ruled out, hence was considered as the second differential.

**Figure 7:**
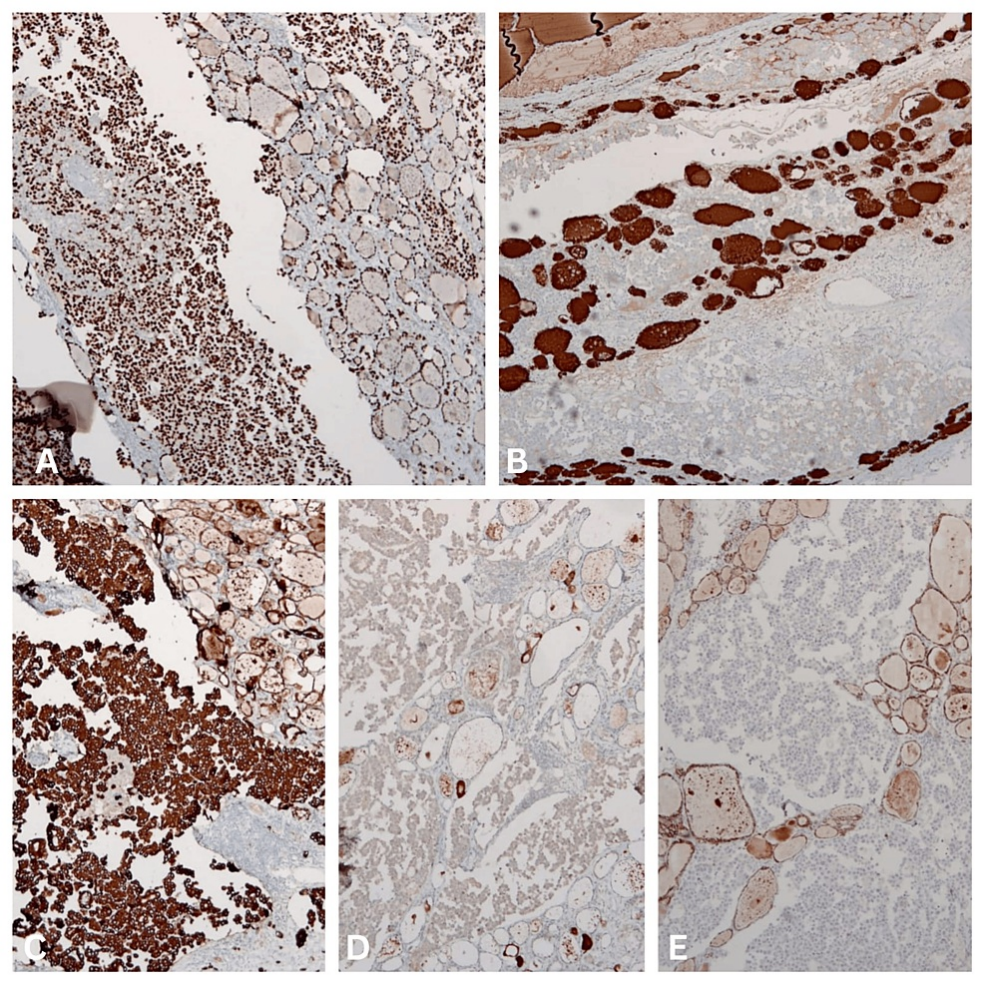
IHC images: Figure (A) showing TTF1 nuclear expression in tumour cells. Figure (B) showing Thyroglobulin positivity in tumour cells. Figure (C) showing CK19 positivity in tumour cells. Figure (D) showing BRAF expression in tumour cells and Figure (E) showing loss of CD56 in tumour cells. IHC: Immunohistochemistry

Eventually, when the immunohistochemical study was conducted, it showed multifocal deposits of a malignant papillary neoplasm comprising round to cuboidal tumour cells having round/oval hyperchromatic nuclei, eosinophilic cytoplasm, with occasional nuclear grooving and chromatin clearing, arranged in a papillary and glandular pattern. The tumour cells exhibited positive staining for CK19, TTF-1, BRAF, and Thyroglobulin, confirming the diagnosis of multifocal deposits of oncocytic papillary thyroid carcinoma (Figure 7).

## Discussion

Various morphological forms of papillary thyroid carcinoma have been recognized through assessments of their architectural characteristics, growth patterns, and cellular as well as stromal attributes [2]. The identification of nuclear attributes, including intermittent grooving and chromatin clearing, along with dense colloid-filled follicles, is also noteworthy. Hence, the possibility of a primary malignancy of the thyroid, most probably a variant of papillary carcinoma thyroid, was considered on histopathological examination. It was contemplated as a differential consideration due to the absence of classical nuclear features like nuclear crowding, ground glass nuclei (Orphan Annie nuclei), frequent nuclear grooves, nuclear pseudoinclusions and psammoma bodies (laminated microcalcifications) [5].

Primary thyroid malignancies typically cause disruption of the normal follicular cell architecture. The maintained integrity of the thyroid follicles, in this case with infiltrating atypical pleomorphic neoplastic cells infiltrating in between these benign follicles, led to a strong suspicion of metastatic deposits, rather than a tumour of primary site origin [6]. CT scans of the thorax indicated the existence of a soft tissue lesion with spiculated borders in the apical segment of the right upper lobe raising the suspicion of a probable neoplastic lesion of pulmonary origin, suggesting metastatic deposits in the thyroid. Furthermore, the glandular and acinar arrangement of the infiltrating tumour cells brought us to the conclusion of metastatic deposits of adenocarcinoma in the thyroid as a key differential diagnosis [7].

This goes on to prove that papillary carcinoma of the thyroid can have a very diverse presentation [8]. Even with the absence of all the classical features, the subtle findings of occasional nuclear grooving and chromatin clearing along with dense colloid-filled follicles should raise suspicion. This unusual case reaffirms the fact that despite all the literature, cancers can indeed present with unique histopathological morphologies that warrant further ancillary molecular pathology research.

## Conclusions

In the realm of thyroid histopathology, arriving at an equivocal diagnosis can be quite challenging. While the microscope has traditionally held a central role in the diagnostic process, pathologists must acknowledge the inherent limitations and, therefore, employ objective markers to distinguish among various closely related high-grade differentiated thyroid carcinomas that necessitate radioactive iodine ablation. This is essential to attain meaningful diagnostic and prognostic outcomes that can effectively alter patient management decisions.
